# An eye-tracking approach to Autonomous sensory meridian response (ASMR): The physiology and nature of tingles in relation to the pupil

**DOI:** 10.1371/journal.pone.0226692

**Published:** 2019-12-26

**Authors:** Niilo V. Valtakari, Ignace T. C. Hooge, Jeroen S. Benjamins, Anouk Keizer

**Affiliations:** 1 Experimental Psychology, Helmholtz Institute, Utrecht University, Utrecht, the Netherlands; 2 Social, Health and Organisational Psychology, Utrecht University, Utrecht, the Netherlands; Tohoku University, JAPAN

## Abstract

Autonomous sensory meridian response (ASMR) is a sensory phenomenon commonly characterized by pleasant tingling sensations arising from the back of the head and accompanied by feelings of relaxation and calmness. Although research has found ASMR to have a distinct physiological pattern with increased skin conductance levels and reduced heart rate, the specific tingles felt in ASMR have not received much investigation. The aim of the present study was to investigate the physiology and characteristics of ASMR further by examining whether experiencing ASMR is visible from the pupil of the eye. A total of 91 participants were recruited and assigned to three different groups based on their experience of ASMR (ASMR vs. non-ASMR vs. unsure). Participants were instructed to watch a control video and an ASMR video and to report any tingling sensations by pressing down a button on the keyboard. Pupil diameter was measured over the duration of both videos using a tower-mounted eye tracker. Data was analyzed on a general level, averaging pupil diameter over each video, as well as on a more specific level, comparing pupil diameter during reported episodes of tingling sensations to pupil diameter outside of those episodes. On the general level, results revealed no significant differences between the groups. On the specific level, however, the tingling sensations experienced in ASMR were found to cause statistically significant increases in pupil diameter, demonstrating that they have a physiological basis. The results of the study further reinforce the credibility of ASMR and suggest that the tingles felt in ASMR are at the very core of the experience itself.

## Introduction

Autonomous sensory meridian response (ASMR) refers to a sensory phenomenon that has received a large amount of popular attention over the past few years. The first scientific study on the topic defined experiencing ASMR as static-like tingling sensations felt on the skin accompanied by a mix of positive feelings and relaxation [1]. The tingling sensations in ASMR are often described as starting from the back of the head, expanding down the spine, and sometimes even moving as far as into the limbs [1]. ASMR is usually not triggered spontaneously, but rather in response to particular types of stimuli, of which most common are whispering, personal attention, crisp sounds, and slow movements [1]. A notable characteristic of ASMR is that not everyone experiences it, and its prevalence among the general population has not yet been thoroughly investigated. When browsing internet sites like YouTube, it is hard to not come by the term ASMR; according to Ahrefs, it is currently the 8th most common keyword searched on YouTube worldwide (as of February 2019) [2]. Both anecdotal reports and various studies have suggested that ASMR can have multiple benefits, ranging from mood improvement to temporary relief of physical pain [1, 3], indicating that it could potentially be used as a therapeutic tool.

ASMR has been studied in relation to various other sensory phenomena, including synesthesia, misophonia, and aesthetic chills. One study found that ASMR participants showed a slightly greater prevalence of synesthesia than the general population [1]. Other studies have found connections to misophonia; half of the participants in a large scale misophonia study reported to experience ASMR [4], while another related study showed that ASMR participants were more likely to demonstrate increased levels of misophonia [5]. In a study on the relationship between ASMR and aesthetic chills, the authors concluded that although more research is needed to determine whether aesthetic chills and ASMR are variations of the same phenomenon, the two phenomena share a number of similarities, and suggested that mindfulness is an essential component in both [6].

Even though ASMR has gained in popularity and research into ASMR has been done to some extent, debate whether it is a real phenomenon still exists. In a study of how expectancy effects influence ASMR, participants previously unfamiliar with ASMR were more likely to experience ASMR after their expectations were manipulated [7]. ASMR participants, however, were not affected by those same manipulations. The researchers concluded that ASMR for some individuals may be a result of expectancy effects; that simply believing a certain type of stimulus to result in a sensory experience will in turn result in that sensory experience, suggesting that at least a part of the ASMR experience could be the result of a placebo effect.

Due to the controversial nature of ASMR, researchers have become increasingly interested in whether the subjective experience of ASMR can be linked to more objective physiological measures. A study by Poerio et al. found that ASMR videos promoted feelings of both calmness and excitement, while reducing heart rate and causing elevations in skin conductance levels [3]. Contrarily, research on aesthetic chills has demonstrated that these awe-inspired experiences are consistently characterized by increased heart rate [8–10], suggesting that the physiological profile of ASMR is distinct from that of aesthetic chills [3]. Similar results were observed in a study involving ASMR and brain imaging; Lochte et al. found that inducing ASMR in participants resulted in increased activity in brain regions typically related to reward activation and emotional arousal, which are also commonly active during aesthetic chills [11]. A key difference in brain activation, however, was that ASMR involved increased activation in regions related to self-awareness, social cognition, and social behavior, whereas these regions usually show decreased activity during aesthetic chills. Taken together, the results of these studies suggest that ASMR is a contradictory experience with a distinct physiological profile, a mixed emotional profile, as well as a social component.

The underlying mechanisms in ASMR are still unknown. The increased activation of brain areas related to social engagement observed in ASMR as well as the commonly social nature of ASMR videos has led to suggestions that ASMR may be similar to social grooming [2, 11], and that the release of oxytocin could be a potential contributor to the tingles [11]. In a study examining the default mode network in relation to ASMR, Smith et al. found that ASMR participants showed significantly reduced functional connectivity in the default mode network (DMN) [12], a similar pattern to that observed in children with attention-deficit/hyperactivity disorder (ADHD) [13], suggesting that a possible explanation for ASMR could be the reduced ability to inhibit sensory-emotional experiences that most people are able to suppress. We think that another way to provide more objective evidence for ASMR as well as further insight into its underlying mechanisms could be to investigate it through the pupil of the eye.

Eye-tracking research has become increasingly popular over the years due to its potential in exploring various different topics as well as its noninvasive nature. As proposed by Daniel Kahneman and later reinforced by a multitude of studies, the pupil of the eye expands in response to increased mental effort [14–15]. Other research on the topic has shown that the pupil is larger in response to both positive and negative emotional stimuli [16–19], and that pupil diameter covaries with skin conductance levels [20]. Increases in pupil diameter have also been linked to other sensory phenomena. A study on the relation of aesthetic chills and pupil diameter revealed that pupil diameter increased significantly during self-reported episodes of musical chills [20]. Upon reviewing literature related to the neuroscience of the pupil response, the researchers concluded that pupil dilation during musical chills is likely a reflection of activation in brain regions collectively known as the locus coeruleus-norepinephrine (LC-NE) system, often described as the “arousal” system of the brain, responsible for the release of the neurotransmitter norepinephrine [20–21]. In a similar study on grapheme-color synesthesia, results showed that pupil diameter was greater for people who experience synesthesia when they were shown incongruently colored symbols reflective of their own synesthetic experience than when the symbols were colored congruently, indicating that the experience of synesthesia is also visible from the pupil [22]. Up to this point, no research combining ASMR with eye tracking exists. Furthermore, only one study so far has investigated ASMR specifically during the actual tingling episodes it is most commonly characterized by [11]. Here we present an eye-tracking approach to examine the specific components of ASMR more closely.

The aim of the present study was to examine the physiological profile of ASMR further. Using eye tracking, we sought to investigate ASMR by measuring pupil diameter while simultaneously inducing it in participants. We wanted to know whether experiencing ASMR in general and during tingling sensations was visible in pupillary changes. In order to accomplish this, we obtained real-time pupil diameter recordings during both an ASMR and a control video from participants who experience ASMR, participants who do not experience ASMR, and participants who are unsure about their ASMR experience. By allowing participants to report tingling sensations via a button press, we were able to further extract recordings of pupil diameter during the actual tingling sensations felt in ASMR. Data was analyzed on a general level, examining pupil diameter over the whole duration of the videos, as well as on a more specific level, examining pupil diameter during the tingles themselves. We expected participants who experience ASMR to show a greater average pupil diameter increase from the control video to the ASMR video when compared to participants who report to not experience ASMR. Moreover, we expected pupil diameter during reported episodes of tingling sensations to be greater when compared to pupil diameter outside of those episodes.

## Materials and methods

### Participants

A total of 91 participants were recruited for the experiment through posters and flyers spread around the campus, social media recruitment channels, and mailing lists. Sixty-three participants were female while 28 were male. Mean age was 25 years with a range of 18 to 60. All participants provided informed consent and were comfortable watching a computer screen at a distance of 57 cm without the need for eyeglasses. Participants were assigned to either the ASMR group, the non-ASMR group, or the unsure group based on their response to the question “Having watched these videos or just from your everyday life, would you classify yourself as someone who experiences ASMR?”. Out of the 91 participants, 37 reported to experience ASMR, 35 reported to not experience ASMR, and 19 reported that they were unsure whether they experience ASMR. The study was approved by the local ethics committee of the Faculty of Social and Behavioural Sciences of Utrecht University (FETC18-089).

### Design

The experiment involved two short videos and a questionnaire for each participant. All participants first viewed the same two videos in a counterbalanced order and then responded to the same questionnaire and were assigned to their respective group after completing the experiment. Participants were measured on three different variables over the course of each video: pupil diameter, the number of tingling sensations, and the total duration of tingling sensations.

### Videos

Two videos were used for the experiment: an ASMR video and a control video. The video used as the ASMR video was a popular ASMR video with over 15 million views on YouTube. It was chosen due to its popularity, static background, static colors, and constant lighting. Permission was obtained by the video creator, and the video was downloaded and edited down to an appropriate length of two minutes. The edited video portrayed a woman providing personal attention toward the viewer in the form of whispering, speaking in a soft voice, hair brushing, and some hand movements. Since different lighting conditions are known to impact pupil diameter [23, 24], we wanted to keep the luminance levels between the ASMR video and the control video as similar as possible. ASMR is predominantly described as an audiovisual experience with both a visual and an auditory component. Because of this, we decided to use the same ASMR video with the audio track removed as the control video, allowing us to keep the luminance of both the ASMR and the control video exactly the same. The original version of the ASMR video we used can be found on YouTube (https://www.youtube.com/watch?v=B8jUVci17vE). In order to further assess pupillary reaction in response to differing levels of lighting, each participant was shown an additional five-second presentation of a static grey, black, and white image.

### Measures

#### Pupil diameter

Pupil diameter was measured using the SensoMotoric Instruments (SMI) Hi-Speed tower-mounted eye tracker. Pupil diameter and gaze position was recorded from the right eye at a sampling rate of 240 Hz for the duration of each video and presentation of the static images. All videos and static images were presented at a distance of 57 cm on a 19-inch HP monitor using a resolution of 1280 x 1024 pixels. Gaze position was recorded in horizontal and vertical coordinates. Pupil diameter was recorded in pixels and converted into millimeters using the recording of a printed-out black circle with a diameter of five millimeters to determine the conversion ratio (1 mm = 17.2 pixels).

#### Tingling sensations

Participants were instructed to press down a button whenever they experienced tingling sensations anywhere in their body during each video. The start point (i.e. when the button was pressed down) and end point (i.e. when the button was released) of each button press was recorded to identify and extract episodes of tingling sensations as well as to count the number and duration of the tingling sensations felt by each participant.

#### Questionnaire

At the end of the experiment, each participant was given a questionnaire with a variety of questions related to their demographic, experience of the videos, and experience of ASMR in general. An overview of responses to the questionnaire is presented in the supporting information (S1 Appendix).

### Procedure

Testing of participants took place at the laboratories of the Department of Psychology at Utrecht University. Each session began with a brief oral explanation of the experiment process provided by the experimenter. After providing informed consent, participants were asked to place their head in an appropriate way on the tower-mounted SMI eye tracker and put on a pair of Bose QuietComfort 25 noise cancelling headphones. Prior to the presentation of the videos, each participant completed a nine-point gaze calibration procedure. After successful calibration, an instruction screen was presented. Participants were instructed to keep their head as still as possible, to refrain from speaking, to fixate their gaze at the center of the screen, and to report any tingling sensations felt anywhere in the body by pressing down a specified button on the keyboard. The instruction screen was followed by a static grey image for a duration of five seconds, after which one of the videos was played. To avoid potential effects that might result from the presentation order of the videos (e.g. participants who saw the control video first might experience the ASMR video in a different way than participants who saw the ASMR video first), the order of the videos was counterbalanced so that half of the participants saw the ASMR video first while the other half saw the control video first. The first video was always followed by a subsequent five-second presentation of a static black image immediately followed by a static white image. The second video was played directly after the static white image. After finishing the second video, participants were asked to fill in a questionnaire with items related to their demographic, experience of the videos, and experience of ASMR in general. Upon completion of the experiment, participants were offered an adequate reward as compensation.

## Results

### Data cleanup and final sample

Pupil data from two participants failed to record due to technical problems with the eye tracker. These participants were excluded from all of the following analyses. For the eye-tracking data of each participant, periods of eye blinking were identified as instances of missing gaze data. Upon examination of the data around these periods, we noticed that the velocity of pupil diameter change was greatly increased in their immediate proximity, likely representing measurement error during the closing and opening of the recorded eye. In attempt to remove these instances of high velocity pupil change, the periods of missing data were expanded on both sides, meaning that four samples of data (equaling to roughly 0.021 seconds with a sampling rate of 240 Hz) both before and after each missing data point were removed. Total data loss percentage for each participant was determined by calculating the amount of data loss due to missing gaze data and adding the additional data loss percentage of the remaining data due to the expansion of missing data points to that value. Participants with over 50 percent data loss were excluded from the analyses. The cleanup process was automated using MATLAB, and all subsequent analyses were conducted using IBM SPSS Statistics 25.0. The final sample used for the analyses consisted of 30 ASMR participants, 33 non-ASMR participants, and 19 unsure participants.

### Gaze fixation and general pupillary response checks

To determine whether participants in the three groups showed different patterns of gaze fixation, the standard deviation of the horizontal and vertical gaze signal for each participant was calculated. A 3 x 2 repeated measures analysis of variance (ANOVA) with group type (ASMR vs. non-ASMR vs. unsure) as the between-subjects variable, video type (ASMR vs. control) as the within-subjects variable, and the standard deviation of the horizontal and vertical signals as the dependent variables did not reveal a significant interaction between video type and group type (*F*(2,156) = 0.64, *p* = .531 for the horizontal signal and *F*(2,156) = 1.29, *p* = .281 for the vertical signal). To further confirm that participants showed similar pupillary responses to different levels of lighting, we ran a 3 x 3 repeated measures ANOVA with group type as the between-subjects variable, static image type (grey vs. black vs. white) as the within-subjects variable, and average pupil diameter during the static images as the dependent variable. A total of four participants did not have data for at least one of the image types and were therefore excluded from the analysis. The interaction between static image type and group type was not found significant, *F*(4,300) = 0.93, *p* = .448.

### Pupil diameter

**Fig 1:**
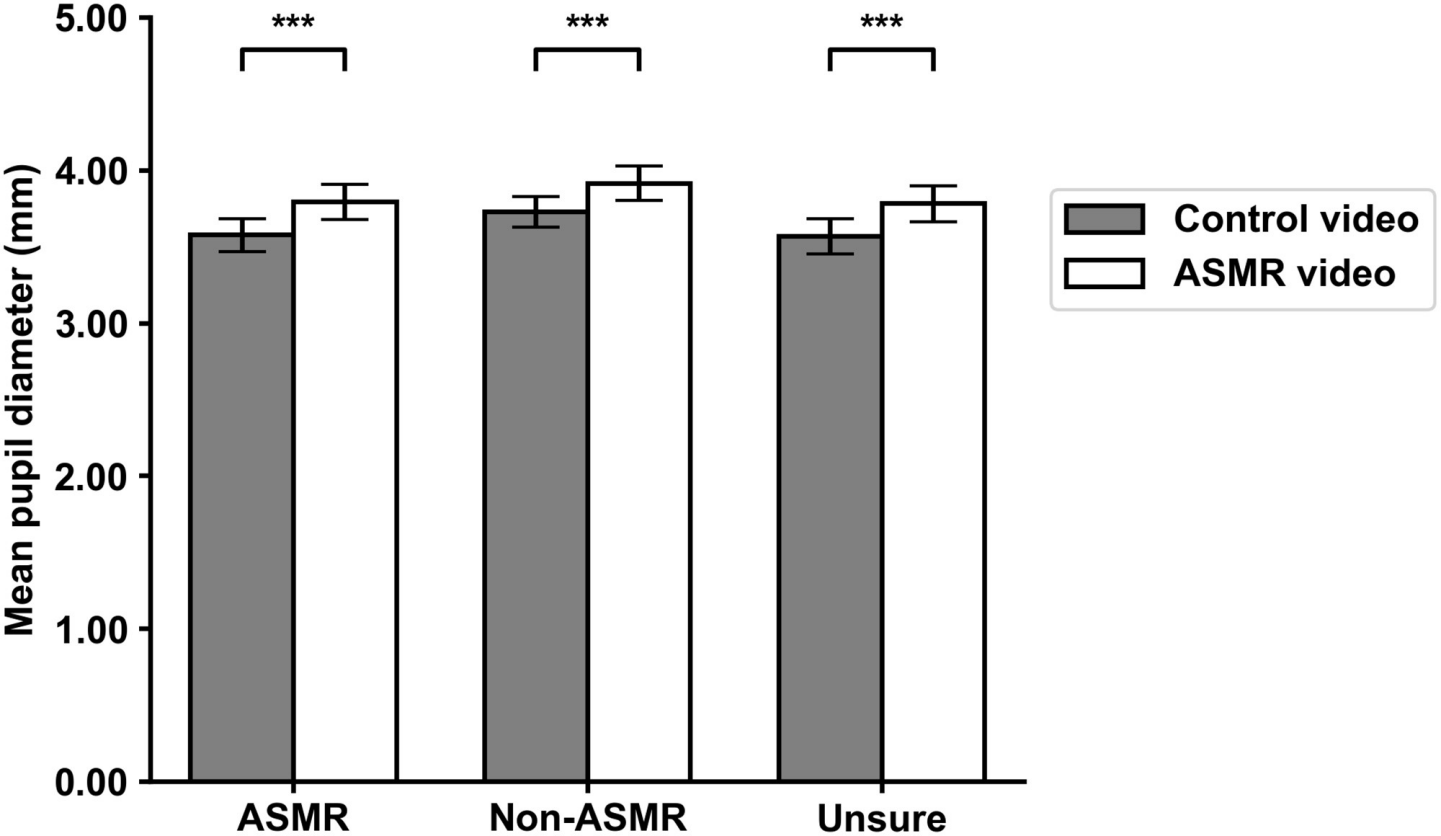
Mean pupil diameters between the videos for all three groups. Bars on top represent standard error. * = *p* < .05, ** = *p* < .01, *** = *p* < .001.

In order to examine pupil diameter between the three different groups and the two videos, we conducted a 3 x 2 repeated measures ANOVA with group type as the between-subjects variable, video type as the within-subjects variable, and pupil diameter as the dependent variable. A significant main effect on video type was observed, *F*(1,79) = 48.07, *p* < .001. Participants in the ASMR group (M_diff_ = -.22, *p* < .001), the non-ASMR group (M_diff_ = -.19, *p* < .001), and the unsure group (M_diff_ = -.21 *p* < .001) all showed significantly greater pupil diameter during the ASMR video. The interaction between group type and video type was not significant, *F*(2,158) = 0.13, *p* = .878. A visualization of these results along with means and standard deviations are presented in Fig 1 and Table 1, respectively.

**Table 1:** Tingling sensations and pupil diameter means for all three groups.

Group	ASMR (N = 30)		Non-ASMR (N = 33)		Unsure (N = 19)	
Video	Control	ASMR	Control	ASMR	Control	ASMR
**Duration of tingles (s)**	1.61 (2.81)	14.49 (13.96)	0.41 (1.67)	0.27 (0.89)	0.83 (2.28)	8.83 (11.61)
**Number of tingles**	2.33 (4.18)	8.77 (9.62)	0.39 (1.62)	0.39 (1.25)	0.42 (1.01)	4.00 (3.68)
**Pupil diameter (mm)**	3.58 (0.59)	3.79 (0.63)	3.73 (0.58)	3.92 (0.64)	3.57 (0.51))	3.78 (0.51)

*Note*. Number of tingles is the number of times a keyboard button was pressed. Duration of tingles is reported as seconds (s). Pupil diameter is reported in millimeters (mm). Values consist of means and standard deviations.

**Fig 2:**
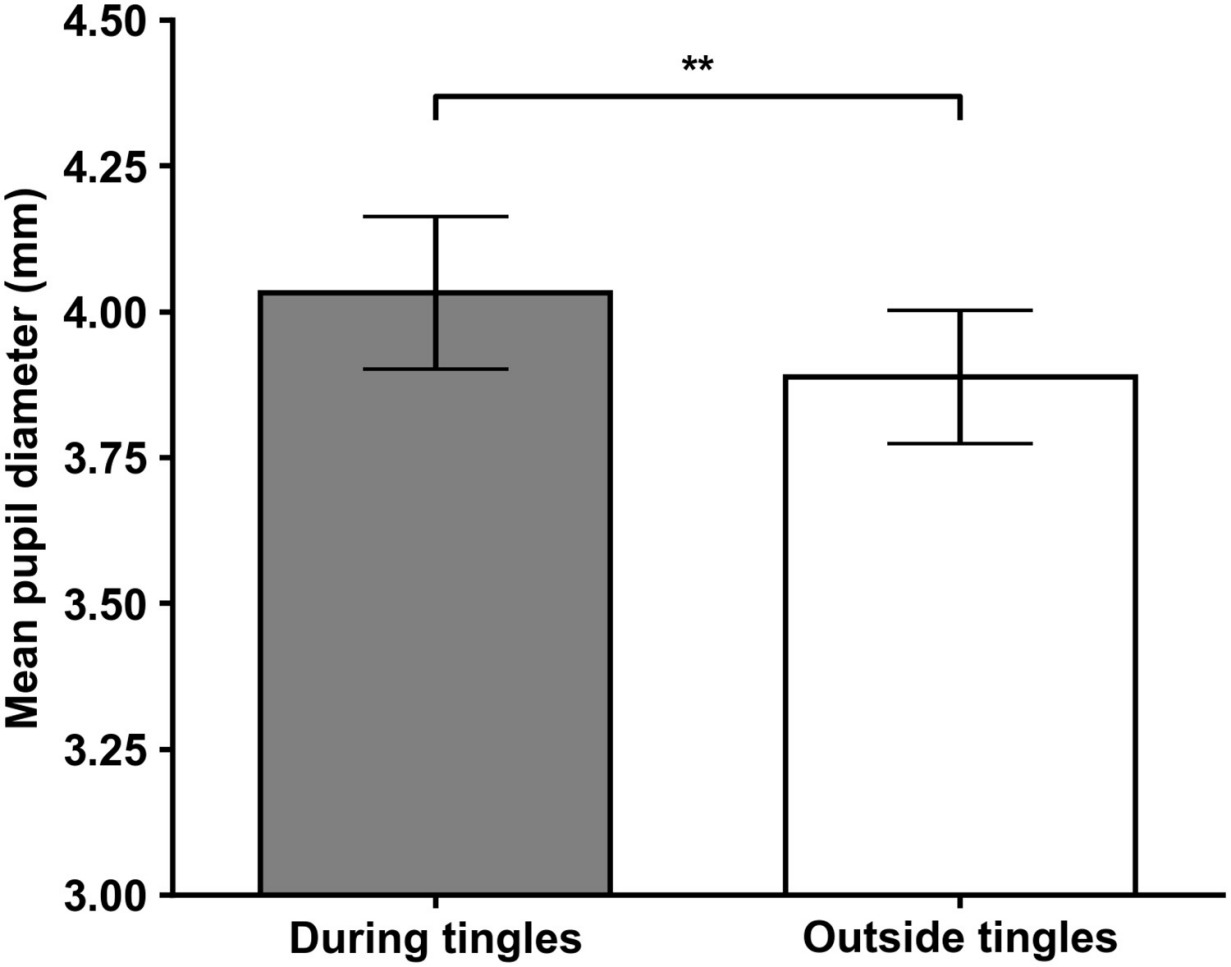
Corrected pupil diameter during tingles compared to pupil diameter outside tingles for ASMR group during ASMR video. Bars on top represent standard error. * = *p* < .05, ** = *p* < .01, *** = *p* < .001.

To examine whether pupil diameter was greater during reported tingling sensations, we ran a paired t-test comparing pupil diameter during reported episodes of tingling sensations to pupil diameter outside of those episodes. Pupil diameter both during and outside of tingling sensations was extracted and averaged for each participant. To determine the effect of the manual response of reporting tingles on pupil diameter, we conducted a follow-up experiment with six participants. The results of the follow-up experiment, which are presented in the supporting information (S1 Appendix), suggested that the manual response accounted for a 0.036 mm increase in pupil diameter. In accordance with these results, a correction of 0.036 mm was applied to the average pupil diameter during reported tingling sensations. To ensure an accurate representation of ASMR, results were only analyzed for the ASMR group and during the ASMR video only. Four participants in the ASMR group did not report tingling sensations during the ASMR video and were therefore excluded from the analysis. The results of this analysis proved significant: pupil diameter was significantly greater during reported episodes of tingling sensations, *t*(25) = 3.61, *p* = .001 (Fig 2). Mean pupil diameters for within tingles and outside of tingles are presented in Table 2.

**Table 2:** Mean pupil diameter during and outside of tingling sensations for ASMR group during ASMR video. (N = 26).

**Pupil diameter during reported tingles**	4.07 (0.67)
**Corrected pupil diameter during reported tingles**	4.03 (0.67)
**Pupil diameter outside reported tingles**	3.89 (0.58)

*Note*. Pupil diameter is reported in millimeters (mm). Values consist of means and standard deviations.

### Tingling sensations

**Fig 3:**
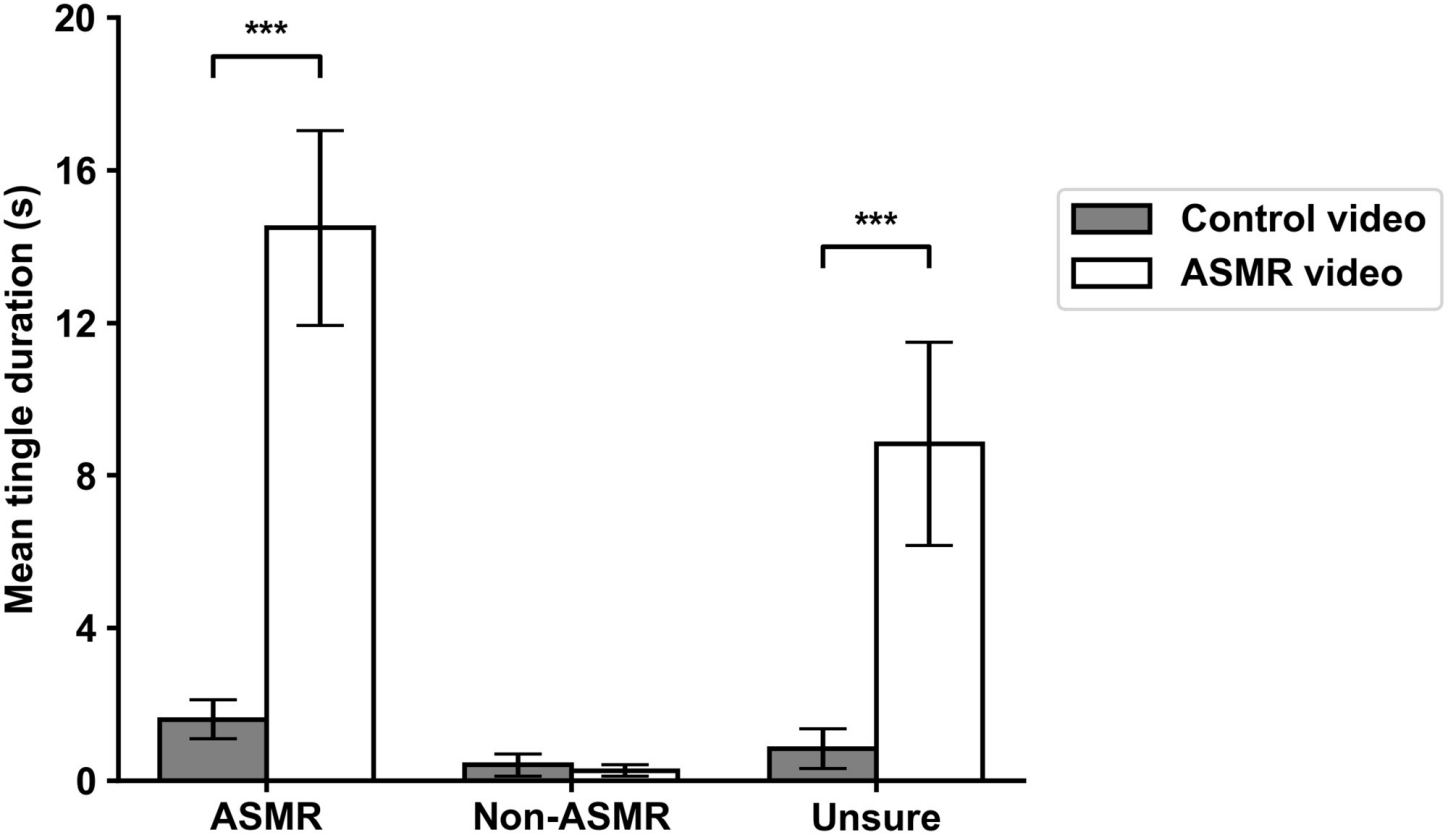
Mean duration of tingling sensations for all three groups. Bars on top represent standard error. * = *p* < .05, ** = *p* < .01, *** = *p* < .001.

**Fig 4:**
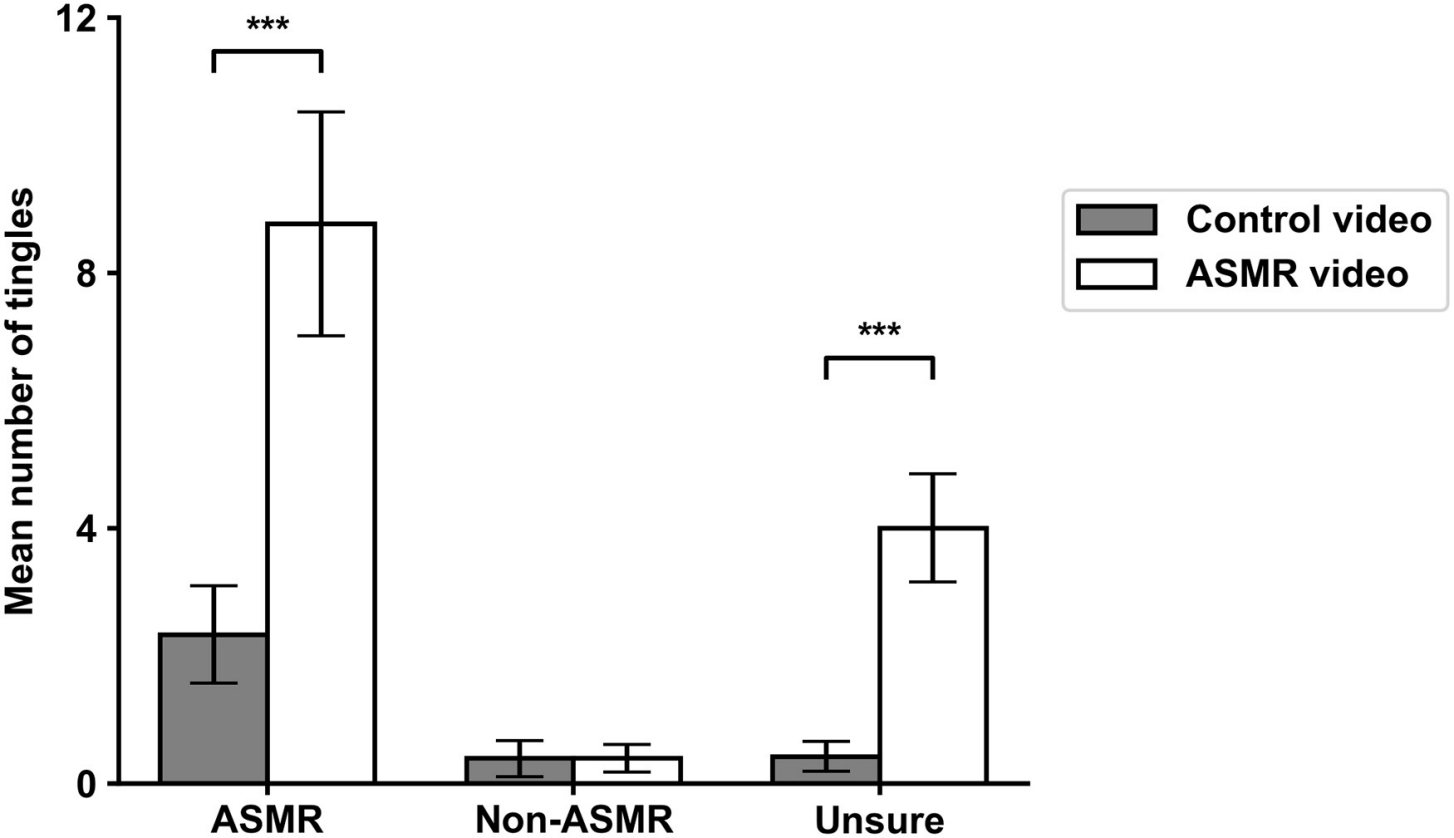
Average number of tingling sensations for all three groups. Bars on top represent standard error. * = *p* < .05, ** = *p* < .01, *** = *p* < .001.

In order to examine the tingles, we calculated the total duration and number of tingling sensations experienced by each participant. A 3 x 2 repeated measures ANOVA with group type as the between-subjects variable and video type as the within-subjects variable revealed a significant interaction between the two variables for the duration of tingling sensations, *F*(2,158) = 16.49, *p* < .001. Participants in the ASMR group (M_diff_ = -12.88 seconds, *p* < .001) and the unsure group (M_diff_ = -7.99 seconds, *p* < .001) reported significantly longer tingle duration for the ASMR video compared to the control video, which was not the case for non-ASMR participants (M_diff_ = -148.21, *p* = .925) (See Fig 3). Similarly, the interaction between group type and video type was significant for the number of tingling sensations, *F*(2,158) = 15.84, *p* < .001; both ASMR (M_diff_ = -6.43 episodes, *p* < .001) and unsure (M_diff_ = -3.58 episodes, *p* = .001) participants experienced significantly more episodes tingling sensations for the ASMR video when compared to the control video, while non-ASMR participants did not (M_diff_ > 0.01 episodes, *p* > .05) (See Fig 4). The means and standard deviations for these data are presented in Table 1.

## Discussion

Previous studies have revealed ASMR to be a complex emotional experience with feelings of both relaxation and calmness as well as excitement. Physiological responses to ASMR-inducing content have shown decreases in heart rate and increases in skin conductance levels, but solely on a general level (i.e. over the whole period of watching an ASMR video) [2]. On a more specific level, tingling sensations in ASMR have so far only been associated with increases in brain activity, particularly in the medial prefrontal cortex, nucleus accumbens, dorsal anterior cingulate cortex, supplementary motor areas, and insula [9]. The primary goal of the present study was to examine whether experiencing ASMR is visible from the pupil on both the general level as well as the specific level. We wanted to know whether ASMR participants would show a greater increase in average pupil diameter between the control video and the ASMR video compared to participants who do not experience ASMR, and whether pupil diameter during reported episodes of tingling sensations was larger when compared to pupil diameter outside of those episodes.

To answer these questions, it was important to first confirm that the ASMR video used in the study resulted in more tingling sensations when compared to the control video for ASMR participants but not for participants who do not experience ASMR. Results of the statistical analyses showed that both the ASMR and unsure group differed significantly on both number and total duration of tingles on the variable video type, indicating that the ASMR video resulted in more tingling sensations for both the ASMR and unsure group, but not for the non-ASMR group. As seen from Figs 3 and 4, the control video resulted in a very minimal amount of tingles for all three groups, whereas the ASMR video resulted in a significant amount of tingles for both the ASMR and unsure groups but not the control group. Important to note, however, is that despite the fact that reports of tingling sensations for the control video were minimal, a small portion of the ASMR group still reported to have experienced tingling sensations during the control video, suggesting that it can be possible to experience ASMR even without the inclusion of sound.

We hypothesized that the increase in pupil diameter from the control video to the ASMR video would be greater for participants who reported to experience ASMR than for participants who reported to not experience ASMR. Notably, all three groups showed a significant increase in pupil diameter between the control and the ASMR video. This increase can likely be explained by the absence of an audio track in the control video; simply presenting an auditory stimulus has been shown to cause increases in pupil diameter [25]. Contrary to our hypothesis, however, the interaction between group type and video type was not significant. One explanation for the absence of an interaction between group type and video type could be that the pupil reacts to ASMR only during the actual tingling sensations. Changes in pupil diameter in reaction to ASMR, even when present, are likely to be quite small; the uncorrected increase in pupil diameter during reported tingling sensations in the present study was only 0.18 mm on average. Furthermore, not all participants in the ASMR group experienced tingling sensations during the ASMR video, and even those who did only experienced them for an average duration of approximately 14.5 seconds. A small increase in pupil diameter during a small portion over the whole duration of the videos is not likely to influence the total average by a large margin. In addition to this, multiple participants reported tingling sensations for both the control video and the ASMR video, further reducing the difference in ASMR experience between the two videos. If pupil diameter only increased during the actual tingles in ASMR, it could potentially explain why average change in pupil diameter between the videos was not significantly different for the three groups.

We not only hypothesized that the increase in pupil diameter between the videos would be greater for ASMR participants, but also that pupil diameter would likely increase during self-reported tingles. To examine tingles on an individual level, we compared mean pupil diameter during reported episodes of tingling sensations to mean pupil diameter outside of reported tingling episodes for each participant. Pupil diameter during reported episodes of tingling sensations was significantly greater when compared to pupil diameter outside of those episodes even when correcting for pupil diameter increase due to the manual response of pressing a button, suggesting that the tingling sensations in ASMR are accompanied by dilation of the pupil. The uncorrected increase in pupil diameter during tingles we observed in our results is similar to that of musical chills [20] and slightly greater than values seen in research on synesthesia [22].

In the present study, we examined the tingling sensations in ASMR further and found them to be accompanied by increases in pupil diameter. We observed a significant difference in pupil diameter only during reported episodes of tingling sensations, but not throughout the entire ASMR video. We see this pattern as reflective of the importance of tingling sensations in ASMR; the whole experience of ASMR might involve multiple components resulting in different reactions, and the tingling sensations are likely at the very core of the experience itself. The relaxing and calming component of ASMR could potentially be a result of a more general effect accompanied by decreases in heart rate, while the more exciting and arousing aspects of ASMR might be a more direct effect of the tingling sensations that is observed in increased skin conductance levels and pupil dilation. Whether or not this is the case, the tingles felt in ASMR seem to be reflected in the pupil. An increased pupil diameter during both ASMR and aesthetic chills add to the already many similarities these two phenomena have between them, suggesting that the release of norepinephrine in the LC-NE system might be involved in ASMR as well. Further research into the concentrations of neurotransmitters during the tingles in ASMR is likely to yield interesting results.

The present study is the first to examine pupil diameter in relation to ASMR and one of the few studies to dissect the ASMR experience into its more specific levels (i.e. the tingling sensations it is often characterized by). We tested a relatively large sample and employed a control group. When interpreting the results, however, some notable limitations should be kept in mind. Although we included a control group and an ASMR group, a third group of participants unsure about their ASMR experience emerged. The unsure group is likely to be a heterogenous group consisting of both ASMR and non-ASMR participants, and not reflective of either population. Consistent with this idea, our results showed that the unsure group placed in between the ASMR and non-ASMR group in terms of their experience of the number and total duration of tingles. The large number of unsure participants is most likely due to ASMR being a still relatively unknown phenomenon: people unfamiliar with it will have a hard time distinguishing whether they actually experience it. Another reason for this could be the inherent indecisiveness of participants who are prone to respond unsure whenever such an option is offered. To complicate things further, ASMR is quite an ambiguous experience without clearly defined criteria; it is not an easy task to determine what counts as a tingle and what does not. To avoid such ambivalent attitudes towards the ASMR experience, future research needs to find ways to define the criteria for ASMR more clearly.

A limitation of the more general analyses of the present study is the auditory difference between the control video and the ASMR video. Although ASMR videos without sound might be viable options as control videos, the effect of sound on the dependent variable needs to be considered carefully. The increase in pupil diameter between the videos that was observed in the present study likely due to sound only could potentially have masked an interaction effect. In situations where the control video needs to be as similar as possible in levels of lighting as the ASMR video, a better option might be to switch the audio track of the ASMR video to an audio track from a different video known to not induce ASMR, or to include background music, which has been suggested to have an inhibitory effect on ASMR [26]. A further limitation of the study relating to the more specific analyses of the tingling sensations is the extent of influence the manual act of pressing a button had on the pupillary response. To correct for this response, we conducted a follow-up experiment and found the effect to be minor at most. Although this response could be considered quite universal and we do not expect our results to be influenced by the small sample size of the follow-up, a larger sample size would allow us to make more robust conclusions. Future research on the tingles in ASMR should take careful consideration of the manual response when reporting tingles and think of ways to better control for it.

By examining ASMR on both a general and specific level, we have provided evidence that the tingling sensations in ASMR are accompanied by reliable changes in physiology. These results were persistent even when corrected for the effect of the manual response. ASMR is most commonly characterized by the presence of static tingling sensations that can spread throughout the body. By providing evidence that the tingling sensations themselves are coupled with changes in physiology, we have found more support for the idea that they are an essential component of ASMR, potentially reflecting the more exciting and arousing aspect of it. This finding gives further credibility to ASMR and particularly the tingles associated with it, suggesting that it is a real phenomenon that warrants further research. By dissecting and examining the components of ASMR further, better understanding of its underlying mechanisms can be obtained. We suggest future researchers to examine the physiological and emotional responses both during and outside of tingles to better understand the mixed physiological and emotional profile of ASMR and to determine whether certain responses are more likely to be associated with certain components of ASMR. With more understanding of the underlying physiological mechanisms of ASMR, its potential use as a therapeutic tool in various different settings will become clearer.

## Supporting information

S1 Appendix Follow-up experiment and questionnaire responses. Results of the follow-up experiment and an overview of the questionnaire responses.
(DOCX)

S1 Dataset CSV data for the main experiment. All data for the main experiment.
(CSV)

S2 Dataset CSV data for the follow-up experiment. All data for the follow-up experiment.
(CSV)

S1 Data Supplement Supplementary data information. Information on each dataset variable.
(DOCX)
