# Supplementary material for: An eye-tracking approach to Autonomous sensory meridian response (ASMR): The physiology and nature of tingles in relation to the pupil
Source: PLoS One. 2019 Dec 26;14(12):e0226692. doi: 10.1371/journal.pone.0226692 (PMC6932793; doi:10.1371/journal.pone.0226692)
Supplement: S1 Appendix — Results of the follow-up experiment and an overview of the questionnaire responses. (DOCX) [file pone.0226692.s001.docx]

**S1 Appendix**

**Follow-up experiment**

To investigate whether the manual act of pressing a button on the keyboard might influence pupil diameter, we conducted an additional experiment. The follow-up experiment took place in the same location, using the same equipment. A total of six participants were recruited. Participants provided informed consent, after which they were placed in the same eye tracker as in the main experiment. The ASMR video of the main experiment was edited so that the fixation cross at the center of the video deviated slightly in orientation for a total of five times during the two-minute duration of the video. The change in orientation lasted for two seconds each time, and the times for when it would happen were randomly generated. Two editions of the video were made with two different sets of five randomly generated times. Half of the participants viewed one edition while the other half viewed the other edition. To emulate the act of pressing the button in response to ASMR, participants were tasked to push down a specified button on the keyboard whenever they saw the fixation cross deviate in orientation during the video. Upon completion of the task, participants were asked to fill in their gender, age, and their answer to the question “Having watched these videos or just from your everyday life, would you classify yourself as someone who experiences ASMR?”. To make sure ASMR was not an influencing factor in the follow-up, we only analyzed data for participants who reported to not experience ASMR. Pupil diameter data during and outside of the button presses was extracted and analyzed for five of the six participants using a paired samples t-test. The results of this analysis were nonsignificant, *t*(4) = .86, *p* = .438. However, we observed that pupil diameter changed 0.036 millimeters on average during the button press episodes and corrected for it in the main results.

**Questionnaire responses**

**Table A. Experience of control video for all three groups.**

|  | ASMR (N = 37) | | | Non-ASMR (N = 35) | | | Unsure (N = 19) | | |
| --- | --- | --- | --- | --- | --- | --- | --- | --- | --- |
|  | Yes | No | Unsure | Yes | No | Unsure | Yes | No | Unsure |
| Did you have an ASMR experience when watching the video? | 7 (19.4) | 24 (66.7) | 5 (13.9) | 0 (0) | 34 (97.1) | 1 (2.9) | 0 (0) | 16 (84.2) | 3 (15.8) |
| If you had an ASMR experience, how did it relate to your other ASMR experiences? |  | | |  | | |  | | |
| Much less intense – Much more intense | 2.00 (1.07) | | | 4.00 (-) | | | 2.50 (2.12) | | |
| Did you experience tingling sensations during the video? |  | | |  | | |  | | |
| None of the time – All of the time | 2.88 (1.25) | | | 5.00 (-) | | | 2.67 (1.53) | | |
| If yes, what was the intensity of the tingling sensations? |  | | |  | | |  | | |
| Not intense at all – Very intense | 2.50 (1.07) | | | 3.00 (-) | | | 2.33 (1.16) | | |
| What did you think about the video you just watched? |  | | |  | | |  | | |
| Did not like it at all -Liked it a lot | 3.03 (1.45) | | | 2.73 (1.42) | | | 2.95 (1.01) | | |
| Did not annoy me at all – Annoyed me a lot | 2.47 (1.53) | | | 3.94 (1.39) | | | 3.63 (1.77) | | |
| Not relaxed and calm at all – Extremely relaxed and calm | 3.80 (1.35) | | | 3.61 (1.14) | | | 3.74 (1.33) | | |
| Distracted and unfocused – Hyperfocused and trance-like | 3.60 (1.30) | | | 3.21 (1.56) | | | 3.42 (1.35) | | |
| Very unsafe – Very safe | 5.00 (1.23) | | | 4.69 (1.26) | | | 4.58 (1.17) | | |

*Note.* Responses for yes/no/unsure questions are reported as frequencies, N (%). Responses for other questions were presented on a seven-point Likert scale, and are reported as mean (SD).

*One participant in the ASMR group was excluded from the frequencies due to missing data.

**Table B. Experience of ASMR video for all three groups.**

|  | ASMR (N = 37) | | | Non-ASMR (N = 35) | | | Unsure (N = 19) | | |
| --- | --- | --- | --- | --- | --- | --- | --- | --- | --- |
|  | Yes | No | Unsure | Yes | No | Unsure | Yes | No | Unsure |
| Did you have an ASMR experience when watching the video? | 25 (69.4) | 6 (16.7) | 5 (13.9) | 0 (0) | 27 (77.1) | 8 (22.9) | 2 (10.5) | 4 (21.1) | 13 (68.4) |
| If you had an ASMR experience, how did it relate to your other ASMR experiences? |  | | |  | | |  | | |
| Much less intense – Much more intense | 3.21 (1.44) | | | 3.8 (1.48) | | | 3.60 (1.27) | | |
| Did you experience tingling sensations during the video? |  | | |  | | |  | | |
| None of the time – All of the time | 4.32 (1.38) | | | 3.00 (1.27) | | | 3.43 (1.22) | | |
| If yes, what was the intensity of the tingling sensations? |  | | |  | | |  | | |
| Not intense at all – Very intense | 3.68 (1.57) | | | 2.33 (0.52) | | | 3.00 (1.04) | | |
| What did you think about the video you just watched? |  | | |  | | |  | | |
| Did not like it at all – Liked it a lot | 5.00 (1.69) | | | 3.49 (1.56) | | | 4.00 (1.63) | | |
| Did not annoy me at all – Annoyed me a lot | 2.09 (1.48) | | | 4.43 (1.58) | | | 3.47 (1.74) | | |
| Not relaxed and calm at all – Extremely relaxed and calm | 5.14 (1.42) | | | 3.89 (1.55) | | | 4.00 (1.25) | | |
| Distracted and unfocused – Hyperfocused and trance-like | 4.53 (1.73) | | | 3.86 (1.17) | | | 4.21 (1.13 | | |
| Very safe – Very unsafe | 5.69 (1.21) | | | 4.69 (1.26) | | | 4.89 (1.37) | | |

*Note.* Responses for yes/no/unsure questions are reported as frequencies, N (%). Responses for other questions were presented on a seven-point Likert scale, and are reported as mean (SD).

*One participant in the ASMR group was excluded from the frequencies due to missing data.

**Table C. Experience of other sensory phenomena for all three groups.**

|  | ASMR (N = 37) | | | Non-ASMR (N = 35) | | | Unsure (N = 19) | | |
| --- | --- | --- | --- | --- | --- | --- | --- | --- | --- |
|  | Yes | No | Unsure | Yes | No | Unsure | Yes | No | Unsure |
| Do you experience misophonia? | 14 (37.8) | 16 (43.2) | 7 (18.9) | 19 (57.6) | 9 (27.3) | 5 (15.2) | 4 (21.1) | 12 (63.2) | 3 (15.8) |
| Do you experience synesthesia? | 4 (10.8) | 25 (67.6) | 8 (21.6) | 1 (3.1) | 26 (81.3) | 5 (14.3) | 2 (10.5) | 13 (68.4) | 4 (21.1) |
| Do you experience aesthetic chills? | 31 (83.8) | 3 (8.1) | 3 (8.1) | 25 (75.8) | 5 (15.2) | 3 (9.1) | 15 (78.9) | 4 (21.1) | 0 (0) |

*Note.* Responses for yes/no/unsure questions are reported as frequencies, N (%).

*Three participants in the non-ASMR group were excluded due to missing data.

**Table D. General descriptive characteristics of ASMR group.**

|  | ASMR (N = 37) | | |
| --- | --- | --- | --- |
|  | Yes | No | Unsure |
| Does the tingling sensation in ASMR originate from the head? | 24 (64.9) | 2 (5.4) | 11 (70.3) |
| Did the experimenter or experimental setting you were in give you ASMR? | 9 (24.3) | 19 (51.4) | 9 (24.3) |
| How often, if at all, do you experience a tingling sensation during an ASMR experience? | | | |
| None of the time – All of the time | 4.76 (1.59) | | |
| What is the intensity of the tingling sensations you feel in general? | | | |
| Not intense at all – Very intense | 4.86 (1.49) | | |
| How do you generally feel during an ASMR experience? | | | |
| Not relaxed and calm at all – Extremely relaxed and calm | 5.81 (1.05) | | |
| Distracted and unfocused – Hyperfocused and trance-like | 5.46 (1.22) | | |
| Not sexually aroused at all – Very sexually aroused | 2.51 (1.64) | | |
| Very uncomfortable – Very comfortable | 5.89 (1.05) | | |
| Very unsafe – Very safe | 5.95 (1.00) | | |

*Note.* Responses for yes/no/unsure questions are reported as frequencies, N (%). Responses for other questions were presented on a seven-point Likert scale, and are reported as mean (SD).
