# Supplementary material for: An eye-tracking approach to Autonomous sensory meridian response (ASMR): The physiology and nature of tingles in relation to the pupil
Source: PLoS One. 2019 Dec 26;14(12):e0226692. doi: 10.1371/journal.pone.0226692 (PMC6932793; doi:10.1371/journal.pone.0226692)
Supplement: S1 Data Supplement — Information on each dataset variable. (DOCX) [file pone.0226692.s004.docx]

| **Main experiment: Variable Information** | | | | |
| --- | --- | --- | --- | --- |
| Variable | Position | Label | Measurement Level | Missing Values |
| ppn | 1 | Participant number | Nominal |  |
| group | 2 | Having watched these videos, or just from your everyday life, would you classify yourself as someone who experiences ASMR? | Nominal | -99 |
| VID_nosound_oridataloss | 3 | VIDEO WITHOUT SOUND - Amount of data loss due to missing gaze data for ASMR video without sound | Scale | -99,000 |
| VID_nosound_interdataloss | 4 | VIDEO WITHOUT SOUND - Amount of data loss due to removal of instances of high velocity pupil diameter change by expanding missing data points for ASMR video without sound | Scale | -99,000 |
| VID_nosound_totaldataloss | 5 | VIDEO WITHOUT SOUND - Total amount of data loss after cleaning up pupil signal (oridataloss + interdataloss) for video without sound | Scale | -99,000 |
| VID_nosound_nofASMRepisodes | 6 | VIDEO WITHOUT SOUND - Number of ASMR episodes as reported by participant (via button press) for ASMR video without sound | Scale | -99 |
| VID_nosound_ASMRdur | 7 | VIDEO WITHOUT SOUND - Total duration of ASMR episodes as reported by participant (via button press) for ASMR video without sound in milliseconds | Scale | -99,000 |
| VID_nosound_AVpupsize | 8 | VIDEO WITHOUT SOUND - Average pupil size for ASMR video without sound in pixels | Scale | -99,000 |
| VID_nosound_AVpupsize_MM | 9 | VIDEO WITHOUT SOUND - Average pupil size for ASMR video without sound in millimeters | Scale | -99,000 |
| VID_nosound_AVpupsizeASMR | 10 | VIDEO WITHOUT SOUND - Average pupil size during reported ASMR episodes for ASMR video without sound in pixels | Scale | -99,000 |
| VID_nosound_AVpupsizeASMR_MM | 11 | VIDEO WITHOUT SOUND - Average pupil size during reported ASMR episodes for ASMR video without sound in millimeters | Scale | -99,000 |
| VID_nosound_AVpupsizeNONASMR | 12 | VIDEO WITHOUT SOUND - Average pupil size outside of reported ASMR episodes for ASMR video without sound in pixels | Scale | -99,000 |
| VID_nosound_AVpupsizeNONASMR_MM | 13 | VIDEO WITHOUT SOUND - Average pupil size outside of reported ASMR episodes for ASMR video without sound in millimeters | Scale | -99,000 |
| VID_nosound_SDx | 14 | VIDEO WITHOUT SOUND - Standard deviation of the horizontal gaze data signal for video without sound in pixels | Scale | -99,00 |
| VID_nosound_SDy | 15 | VIDEO WITHOUT SOUND - Standard deviation of the vertical gaze data signal for video without sound in pixels | Scale | -99,00 |
| VID_sound_oridataloss | 16 | VIDEO WITH SOUND - Amount of data loss due to missing gaze data for ASMR video with sound | Scale | -99,000 |
| VID_sound_interdataloss | 17 | VIDEO WITH SOUND - Amount of data loss due to removal of instances of high velocity pupil diameter change by expanding missing data points for ASMR video with sound | Scale | -99,000 |
| VID_sound_totaldataloss | 18 | VIDEO WITH SOUND - Total amount of data loss after cleaning up pupil signal (oridataloss + interdataloss) for video with sound | Scale | -99,000 |
| VID_sound_nofASMRepisodes | 19 | VIDEO WITH SOUND - Number of ASMR episodes as reported by participant (via button press) for ASMR video with sound | Scale | -99 |
| VID_sound_ASMRdur | 20 | VIDEO WITH SOUND - Total duration of ASMR episodes as reported by participant (via button press) for ASMR video with sound in milliseconds | Scale | -99,000 |
| VID_sound_AVpupsize | 21 | VIDEO WITH SOUND - Average pupil size for ASMR video with sound in pixels | Scale | -99,000 |
| VID_sound_AVpupsize_MM | 22 | VIDEO WITH SOUND - Average pupil size for ASMR video with sound in millimeters | Scale | -99,000 |
| VID_sound_AVpupsizeASMR | 23 | VIDEO WITH SOUND - Average pupil size during reported ASMR episodes for ASMR video with sound in pixels | Scale | -99,000 |
| VID_sound_AVpupsizeASMR_MM | 24 | VIDEO WITH SOUND - Average pupil size during reported ASMR episodes for ASMR video with sound in millimeters | Scale | -99,000 |
| VID_sound_AVpupsizeASMR_MM_CORRECTED | 25 | VIDEO WITH SOUND - Corrected average pupil size during reported ASMR episodes for ASMR video with sound in millimeters (due to manual response) | Scale | -99,00 |
| VID_sound_AVpupsizeNONASMR | 26 | VIDEO WITH SOUND - Average pupil size outside of reported ASMR episodes for ASMR video with sound in pixels | Scale | -99,000 |
| VID_sound_AVpupsizeNONASMR_MM | 27 | VIDEO WITH SOUND - Average pupil size outside of reported ASMR episodes for ASMR video with sound in millimeters | Scale | -99,000 |
| VID_sound_SDx | 28 | VIDEO WITH SOUND - Standard deviation of the horizontal gaze data signal for video with sound in pixels | Scale | -99,00 |
| VID_sound_SDy | 29 | VIDEO WITH SOUND - Standard deviation of the vertical gaze data signal for video with sound in pixels | Scale | -99,00 |
| VID_greyscreen_oridataloss | 30 | GREYSCREEN - Amount of data loss due to missing gaze data for greyscreen video | Scale | -99,000 |
| VID_greyscreen_interdataloss | 31 | GREYSCREEN - Amount of data loss due to removal of instances of high velocity pupil diameter change by expanding missing data points for greyscreen video | Scale | -99,000 |
| VID_greyscreen_totaldataloss | 32 | GREYSCREEN - Total amount of data loss after cleaning up pupil signal (oridataloss + interdataloss) for greyscreen video | Scale | -99,000 |
| VID_greyscreen_AVpupsize | 33 | GREYSCREEN - Average pupil size for greyscreen video in pixels | Scale | -99,000 |
| VID_greyscreen_AVpupsize_MM | 34 | GREYSCREEN - Average pupil size for greyscreen video in millimeters | Scale | -99,000 |
| VID_whitescreen_oridataloss | 35 | WHITESCREEN - Amount of data loss due to missing gaze data for whitescreen video | Scale | -99,000 |
| VID_whitescreen_interdataloss | 36 | WHITESCREEN - Amount of data loss due to removal of instances of high velocity pupil diameter change by expanding missing data points for whitescreen video | Scale | -99,000 |
| VID_whitescreen_totaldataloss | 37 | WHITESCREEN - Total amount of data loss after cleaning up pupil signal (oridataloss + interdataloss) for whitescreen video | Scale | -99,000 |
| VID_whitescreen_AVpupsize | 38 | WHITESCREEN - Average pupil size for whitescreen video in pixels | Scale | -99,000 |
| VID_whitescreen_AVpupsize_MM | 39 | WHITESCREEN - Average pupil size for whitescreen video in millimeters | Scale | -99,000 |
| VID_blackscreen_oridataloss | 40 | BLACKSCREEN - Amount of data loss due to missing gaze data for blackscreen video | Scale | -99,000 |
| VID_blackscreen_interdataloss | 41 | BLACKSCREEN - Amount of data loss due to removal of instances of high velocity pupil diameter change by expanding missing data points for blackscreen video | Scale | -99,000 |
| VID_blackscreen_totaldataloss | 42 | BLACKSCREEN - Total amount of data loss after cleaning up pupil signal (oridataloss + interdataloss) for blackscreen video | Scale | -99,000 |
| VID_blackscreen_AVpupsize | 43 | BLACKSCREEN - Average pupil size for blackscreen video in pixels | Scale | -99,000 |
| VID_blackscreen_AVpupsize_MM | 44 | BLACKSCREEN - Average pupil size for blackscreen video in millimeters | Scale | -99,000 |
| age | 45 | Age of participant | Scale | -99 |
| gender | 46 | Gender of participant | Nominal | -99 |
| PVQ_sound_like | 47 | VIDEO WITH SOUND - What did you think about the video you just watched? ‎/Liking | Ordinal | -99 |
| PVQ_sound_annoy | 48 | VIDEO WITH SOUND - What did you think about the video you just watched? ‎/Annoyance | Ordinal | -99 |
| PVQ_sound_relax | 49 | VIDEO WITH SOUND - How did you feel when watching the video? ‎/Relaxation and calmness | Ordinal | -99 |
| PVQ_sound_focus | 50 | VIDEO WITH SOUND - How did you feel when watching the video? /Focus | Ordinal | -99 |
| PVQ_sound_safe | 51 | VIDEO WITH SOUND - How did you feel when watching the video? /Safety | Ordinal | -99 |
| PVQ_sound_ASMR | 52 | VIDEO WITH SOUND - Did you have an ASMR experience when watching the video? | Nominal | -99 |
| PVQ_sound_ASMRrelate | 53 | VIDEO WITH SOUND - If you had an ASMR experience, how did it relate to your other ASMR experiences? | Ordinal | -99 |
| PVQ_sound_ASMRtingling | 54 | VIDEO WITH SOUND - Did you experience tingling sensations during the video? | Ordinal | -99 |
| PVQ_sound_ASMRintensity | 55 | VIDEO WITH SOUND - If yes, what was the intensity of the tingling sensations | Ordinal | -99 |
| PVQ_nosound_like | 56 | VIDEO WITHOUT SOUND - What did you think about the video you just watched? ‎/Liking | Ordinal | -99 |
| PVQ_nosound_annoy | 57 | VIDEO WITHOUT SOUND - What did you think about the video you just watched? ‎/Annoyance | Ordinal | -99 |
| PVQ_nosound_relax | 58 | VIDEO WITHOUT SOUND - How did you feel when watching the video? ‎/Relaxation and calmness | Ordinal | -99 |
| PVQ_nosound_focus | 59 | VIDEO WITHOUT SOUND - How did you feel when watching the video? /Focus | Ordinal | -99 |
| PVQ_nosound_safe | 60 | VIDEO WITHOUT SOUND - How did you feel when watching the video? /Safety | Ordinal | -99 |
| PVQ_nosound_ASMR | 61 | VIDEO WITHOUT SOUND - Did you have an ASMR experience when watching the video? | Nominal | -99 |
| PVQ_nosound_ASMRrelate | 62 | VIDEO WITHOUT SOUND - If you had an ASMR experience, how did it relate to your other ASMR experiences? | Ordinal | -99 |
| PVQ_nosound_ASMRtingle | 63 | VIDEO WITHOUT SOUND - Did you experience tingling sensations during the video? | Ordinal | -99 |
| PVQ_nosound_ASMRintensity | 64 | VIDEO WITHOUT SOUND - If yes, what was the intensity of the tingling sensations | Ordinal | -99 |
| AD01_tinglefrequency | 65 | How often, if at all, do you experience a tingling sensation during an ASMR experience? | Ordinal | -99 |
| AD02_tingleorigin | 66 | Does this tingling sensation originate from the head? | Nominal | -99 |
| AD03_tingleintensity | 67 | What is the intensity of the tingling sensations? | Ordinal | -99 |
| AD04_relax | 68 | How do you generally feel during an ASMR experience? ‎/Relaxation and calmness | Ordinal | -99 |
| AD05_focus | 69 | How do you generally feel during an ASMR experience? ‎/Focus | Ordinal | -99 |
| AD06_sexualarousal | 70 | How do you generally feel during an ASMR experience? ‎/Sexual arousal | Ordinal | -99 |
| AD07_comfort | 71 | How do you generally feel during an ASMR experience? ‎/Comfort | Ordinal | -99 |
| AD08_safe | 72 | How do you generally feel during an ASMR experience? ‎/Safety | Ordinal | -99 |
| AD09_previous | 73 | Prior to this experiment, approximately how many days has it been since your previous ASMR experience?‎ | Scale | -99 |
| AD10_touch | 74 | Although you are not being touched during an ASMR experience, does it seem as if you feel sensations on your skin? | Ordinal | -99 |
| AD11_vidsearch | 75 | Do you specifically search for and watch ASMR videos online? | Ordinal | -99 |
| AD12_difference | 76 | If you search for and watch ASMR videos online, do you experience a difference between ASMR experiences triggered by a video compared to ASMR experiences triggered by real life situations? | Ordinal | -99 |
| AD13_embarassed | 77 | Do you feel embarassed by your ASMR experiences? | Ordinal | -99 |
| AD14_family | 78 | Do you know or suspect any of your family members to have ASMR experiences as well? | Nominal | -99 |
| AD15_familyexplain | 79 | Do you know or suspect any of your family members to have ASMR experiences as well? - please explain your answer | Nominal |  |
| AD16_ageonset | 80 | At what age did you have your first ASMR experience? | Nominal | -99 |
| AD17_top3triggers | 81 | Can you briefly describe your top 3 triggers (e.g. "whispering" or "crunchy sounds")? | Nominal |  |
| AD18_misophonia | 82 | Do you experience misophonia? | Nominal | -99 |
| AD19_misophoniatrigger | 83 | Can you shortly describe your strongest misophonia trigger? | Nominal |  |
| AD20_synesthesia | 84 | Do you experience synesthesia? | Nominal | -99 |
| AD21_synesthesiatype | 85 | Can you shortly describe which type of synesthesia you have? | Nominal |  |
| AD22_aesthetic | 86 | Do you have aesthetic experiences of awe which often involve shivers or goosebumps in response to for example a piece of music or art that you really enjoy? | Nominal | -99 |
| AD23_experimenter | 87 | Did the experimenter or experimental setting you were just in give you ASMR? | Nominal | -99 |

| **Main experiment: Variable Values** | | |
| --- | --- | --- |
| Value | | Label |
| group | 1 | yes |
|  | 2 | no |
|  | 3 | unsure |
| VID_nosound_oridataloss | ,000 | no data loss |
|  | 1,000 | complete data loss |
| VID_nosound_interdataloss | ,000 | no data loss |
|  | 1,000 | complete data loss |
| VID_nosound_totaldataloss | ,000 | no data loss |
|  | 1,000 | complete data loss |
| VID_sound_oridataloss | ,000 | no data loss |
|  | 1,000 | complete data loss |
| VID_sound_interdataloss | ,000 | no data loss |
|  | 1,000 | complete data loss |
| VID_sound_totaldataloss | ,000 | no data loss |
|  | 1,000 | complete data loss |
| VID_greyscreen_oridataloss | ,000 | no data loss |
|  | 1,000 | complete data loss |
| VID_greyscreen_interdataloss | ,000 | no data loss |
|  | 1,000 | complete data loss |
| VID_greyscreen_totaldataloss | ,000 | no data loss |
|  | 1,000 | complete data loss |
| VID_whitescreen_oridataloss | ,000 | no data loss |
|  | 1,000 | complete data loss |
| VID_whitescreen_interdataloss | ,000 | no data loss |
|  | 1,000 | complete data loss |
| VID_whitescreen_totaldataloss | ,000 | no data loss |
|  | 1,000 | complete data loss |
| VID_blackscreen_oridataloss | ,000 | no data loss |
|  | 1,000 | complete data loss |
| VID_blackscreen_interdataloss | ,000 | no data loss |
|  | 1,000 | complete data loss |
| VID_blackscreen_totaldataloss | ,000 | no data loss |
|  | 1,000 | complete data loss |
| gender | 1 | Male |
|  | 2 | Female |
| PVQ_sound_like | 1 | Did not like it at all |
|  | 7 | Liked it a lot |
| PVQ_sound_annoy | 1 | Did not annoy me at all |
|  | 7 | Annoyed me a lot |
| PVQ_sound_relax | 1 | Not relaxed and calm at all |
|  | 7 | Extremely relaxed and calm |
| PVQ_sound_focus | 1 | Distracted and unfocused |
|  | 7 | Hyperfocused and trance-like |
| PVQ_sound_safe | 1 | Very unsafe |
|  | 7 | Very safe |
| PVQ_sound_ASMR | 1 | yes |
|  | 2 | no |
|  | 3 | unsure |
| PVQ_sound_ASMRrelate | 1 | Much less intense |
|  | 7 | Much more intense |
| PVQ_sound_ASMRtingling | 1 | None of the time |
|  | 7 | All of the time |
| PVQ_sound_ASMRintensity | 1 | Not intense at all |
|  | 7 | Very intense |
| PVQ_nosound_like | 1 | Did not like it at all |
|  | 7 | Liked it a lot |
| PVQ_nosound_annoy | 1 | Did not annoy me at all |
|  | 7 | Annoyed me a lot |
| PVQ_nosound_relax | 1 | Not relaxed and calm at all |
|  | 7 | Extremely relaxed and calm |
| PVQ_nosound_focus | 1 | Distracted and unfocused |
|  | 7 | Hyperfocused and trance-like |
| PVQ_nosound_safe | 1 | Very unsafe |
|  | 7 | Very safe |
| PVQ_nosound_ASMR | 1 | yes |
|  | 2 | no |
|  | 3 | unsure |
| PVQ_nosound_ASMRrelate | 1 | Much less intense |
|  | 7 | Much more intense |
| PVQ_nosound_ASMRtingle | 1 | None of the time |
|  | 7 | All of the time |
| PVQ_nosound_ASMRintensity | 1 | Not intense at all |
|  | 7 | Very intense |
| AD01_tinglefrequency | 1 | None of the time |
|  | 7 | All of the time |
| AD02_tingleorigin | 1 | yes |
|  | 2 | no |
|  | 3 | unsure |
| AD03_tingleintensity | 1 | Not intense at all |
|  | 7 | Very intense |
| AD04_relax | 1 | Not relaxed and calm at all |
|  | 7 | Extremely relaxed and calm |
| AD05_focus | 1 | Distracted and unfocused |
|  | 7 | Hyperfocused and trance-like |
| AD06_sexualarousal | 1 | Not sexually aroused at all |
|  | 7 | Very sexually aroused |
| AD07_comfort | 1 | Very uncomfortable |
|  | 7 | Very comfortable |
| AD08_safe | 1 | Very unsafe |
|  | 7 | Very safe |
| AD10_touch | 1 | No sensations at all |
|  | 7 | Many sensations |
| AD11_vidsearch | 1 | Never |
|  | 7 | All the time |
| AD12_difference | 1 | Video experiences are much less intense |
|  | 7 | Video experiences are much more intense |
| AD13_embarassed | 1 | Never |
|  | 7 | All the time |
| AD14_family | 1 | yes |
|  | 2 | no |
|  | 3 | unsure |
| AD16_ageonset | 1 | Early childhood |
|  | 2 | Childhood |
|  | 3 | Teenage years |
|  | 4 | Adulthood |
| AD18_misophonia | 1 | yes |
|  | 2 | no |
|  | 3 | unsure |
| AD20_synesthesia | 1 | yes |
|  | 2 | no |
|  | 3 | unsure |
| AD22_aesthetic | 1 | yes |
|  | 2 | no |
|  | 3 | unsure |
| AD23_experimenter | 1 | yes |
|  | 2 | no |
|  | 3 | unsure |

| **Follow-up experiment: Variable Information** | | | | |
| --- | --- | --- | --- | --- |
| Variable | Position | Label | Measurement Level | Missing Values |
| ppn | 1 | Participant number | Nominal | -99 |
| group | 2 | Having watched these videos, or just from your everyday life, would you classify yourself as someone who experiences ASMR? | Nominal | -99 |
| AVpupsize | 3 | Average pupil size during video in pixels | Scale | -99,000000000000000 |
| AVpupsize_button | 4 | Average pupil size during button press episodes in pixels | Scale | -99,000000000000000 |
| AVpupsize_MM | 5 | Average pupil size during video in millimeters | Scale | -99,000000000000000 |
| AVpupsize_buttonMM | 6 | Average pupil size during button press episodes in millimeters | Scale | -99,000000000000000 |
| AVpupsize_outside | 7 | Average pupil size outside button press episodes in pixels | Scale | -99,000000000000000 |
| AVpupsize_outside_MM | 8 | Average pupil size outside button press episodes in millimeters | Scale | -99,000000000000000 |
| button_duration | 9 | Total duration of button press episodes in milliseconds | Scale | -99,0000000000000 |
| nofbuttonpresses | 10 | Total number of button presses during video | Scale | -99 |
| interdataloss | 11 | Amount of data loss due to missing gaze data for video | Scale | -99,000 |
| oridataloss | 12 | Amount of data loss due to expansion of missing data points in attempt to remove instances of high velocity pupil change | Scale | -99,000000000000000 |
| totaldataloss | 13 | Total data loss (oridataloss and interdataloss) | Scale | -99,000000000000000 |
| gender | 14 | Gender of participant | Nominal | -99 |
| age | 15 | Age of participant | Scale | -99 |

| **Follow-up experiment: Variable Values** | | |
| --- | --- | --- |
| Value | | Label |
| group | 1 | yes |
|  | 2 | no |
|  | 3 | unsure |
| gender | 1 | Male |
|  | 2 | Female |
